# The association of hyperketonemia with fecal and rumen microbiota at time of diagnosis in a case-control cohort of early lactation cows

**DOI:** 10.1186/s12917-022-03500-4

**Published:** 2022-11-21

**Authors:** Asha M. Miles, Jessica A. A. McArt, Svetlana F. Lima, Rafael C. Neves, Erika Ganda

**Affiliations:** 1 Department of Animal Science, College of Agricultural Sciences, The Pennsylvania State University, University Park, State College, PA 16802 USA; 2 Current address: Animal Genomics and Improvement Laboratory, Agricultural Research Service, United States Department of Agriculture (USDA), Beltsville, MD 20705 USA; 3 Department of Population Medicine and Diagnostic Sciences, College of Veterinary Medicine, Cornell University, Ithaca, NY 14853 USA; 4 Department of Medicine, Jill Roberts Institute for IBD Research, Weill Cornell Medicine, New York, NY 10021 USA; 5 Department of Veterinary Clinical Sciences, College of Veterinary Medicine, Purdue University, Lafayette, IN 47907 USA

**Keywords:** Dairy cow, Hyperketonemia, Metagenomics, Gut microbiome, 16S rRNA sequencing

## Abstract

**Background:**

Many dairy cows experience a state of energy deficit as they transition from late gestation to early lactation. The aims of this study were to 1) determine if the development of hyperketonemia in early lactation dairy cows is indicated by their gut microbiome, and 2) to identify microbial features which may inform health status. We conducted a prospective nested case-control study in which cows were enrolled 14 to 7 days before calving and followed through their first 14 days in milk (DIM). Hyperketonemic cows (HYK, *n* = 10) were classified based on a blood β-hydroxybutyrate (BHB) concentration 1.2 mmol/L within their first 14 DIM. For each HYK cow, two non-HYK (CON, *n* = 20) cows were matched by parity and 3 DIM, with BHB < 1.2 mmol/L. Daily blood BHB measures were used to confirm CON cows maintained their healthy status; some CON cows displayed BHB 1.2 mmol/L after matching and these cows were reclassified as control-HYK (C-HYK, *n* = 9). Rumen and fecal samples were collected on the day of diagnosis or matching and subjected to 16S rRNA profiling.

**Results:**

No differences in taxa abundance, or alpha and beta diversity, were observed among CON, C-HYK, and HYK health groups for fecal microbiomes. Similar microbiome composition based on beta diversity analysis was detected for all health statuses, however the rumen microbiome of CON and HYK cows were found to be significantly different. Interestingly, highly similar microbiome composition was observed among C-HYK cow rumen and fecal microbiomes, suggesting that these individual animals which initially appear healthy with late onset of hyperketonemia were highly similar to each other. These C-HYK cows had significantly lower abundance of *Ruminococcus* 2 in their rumen microbiome compared to CON and HYK groups. Multinomial regressions used to compute log-fold changes in microbial abundance relative to health status were not found to have predictive value, therefore were not useful to identify the role of certain microbial features in predicting health status.

**Conclusions:**

Lower relative abundance of *Ruminococcus* 2 in C-HYK cow rumens was observed, suggesting these cows may be less efficient at degrading cellulose although the mechanistic role of *Ruminococcus* spp. in rumen metabolism is not completely understood. Substantial differences in fecal or rumen microbiomes among cows experiencing different levels of energy deficit were not observed, suggesting that hyperketonemia may not be greatly influenced by gut microbial composition, and vice versa. Further studies using higher resolution -omics approaches like meta-transcriptomics or meta-proteomics are needed to decipher the exact mechanisms at play.

**Supplementary Information:**

The online version contains supplementary material available at 10.1186/s12917-022-03500-4.

## Background

Dairy cows commonly experience a state of energy deficit as they transition from late gestation to early lactation, a result of both an increase in energy requirements due to milk production and a decrease in dry matter intake. Several animal health and productivity issues emerge as consequence of this energy imbalance including reduced fertility, compromised immune responses, poor feed efficiency, and diminished milk yield [1–4]. To adapt to this energy deficit, cows mobilize lipid reserves which circulate in the blood as non-esterified fatty acids (NEFA). Circulating NEFA can then be used directly as a fuel source, metabolized in the liver to ketone bodies (e.g. β-hydroxybutyrate), or converted into triglycerides. When the liver is overwhelmed by NEFA, ketone bodies are produced in excess and the cow becomes hyperketonemic [5]. In many ways, this transitional period of negative energy balance in dairy cattle is similar to diabetes in humans.

Recent studies have investigated of the role of gut microbiota in energy metabolism and metabolic disease in humans and rat models. Wen et al. (2008) found that mice harboring a defined microbial consortium were significantly affected with Type 1 diabetes [6]. This tight relationship between the gut microbiome and various metabolic disorders, including obesity, diabetes, and cardiovascular diseases has been extensively explored in humans [7, 8], which was found to impact the host innate immune system and energy metabolism in response to microbial derived metabolites and cellular structures. According to the authors, gut microbiota affect the host by modulating its innate immune system and energy metabolism through response to bacterial compounds and bacterial metabolites of dietary compounds. Murri et al. (2013) demonstrated that children with Type 1 diabetes had significantly increased numbers of bacteria of the genus *Clostridium* and *Veillonella* and decreased numbers of *Lactobacillus*, *Bifidobacterium*, the *Blautia coccoides*/*Eubacterium* rectal group and *Prevotella* genus compared to healthy children [9]. Interestingly, the numbers of lactic- and butyric-acid producing bacteria, as well as mucin-degrading bacteria that are shown to be essential for gut integrity were significantly reduced in children with Type 1 diabetes.

In cattle, Oikonomou et al. (2013) found that milk-fed calves with a higher fecal prevalence of *Faecalibacterium* spp. have significantly higher body weight gain during the pre-weaning period [10]. However, a study comparing the composition of the bacterial community and concentration of volatile fatty acids in the rumen during the transition period in dairy cows found no difference in rumen microbiota [11]. Note, the design of that study did not allow proper diagnosis of hyperketonemia in enrolled cows and thus the results must be interpreted with caution. Therefore, in this study we hypothesized that the gut microbiome significantly affects energy metabolism in dairy cows during the transition period. Here we aim to 1) determine whether the development of hyperketonemia in early lactation dairy cows is indicated by their gut microbiome, and 2) to identify microbial features which may inform health status.

## Results

**Fig. 1:**
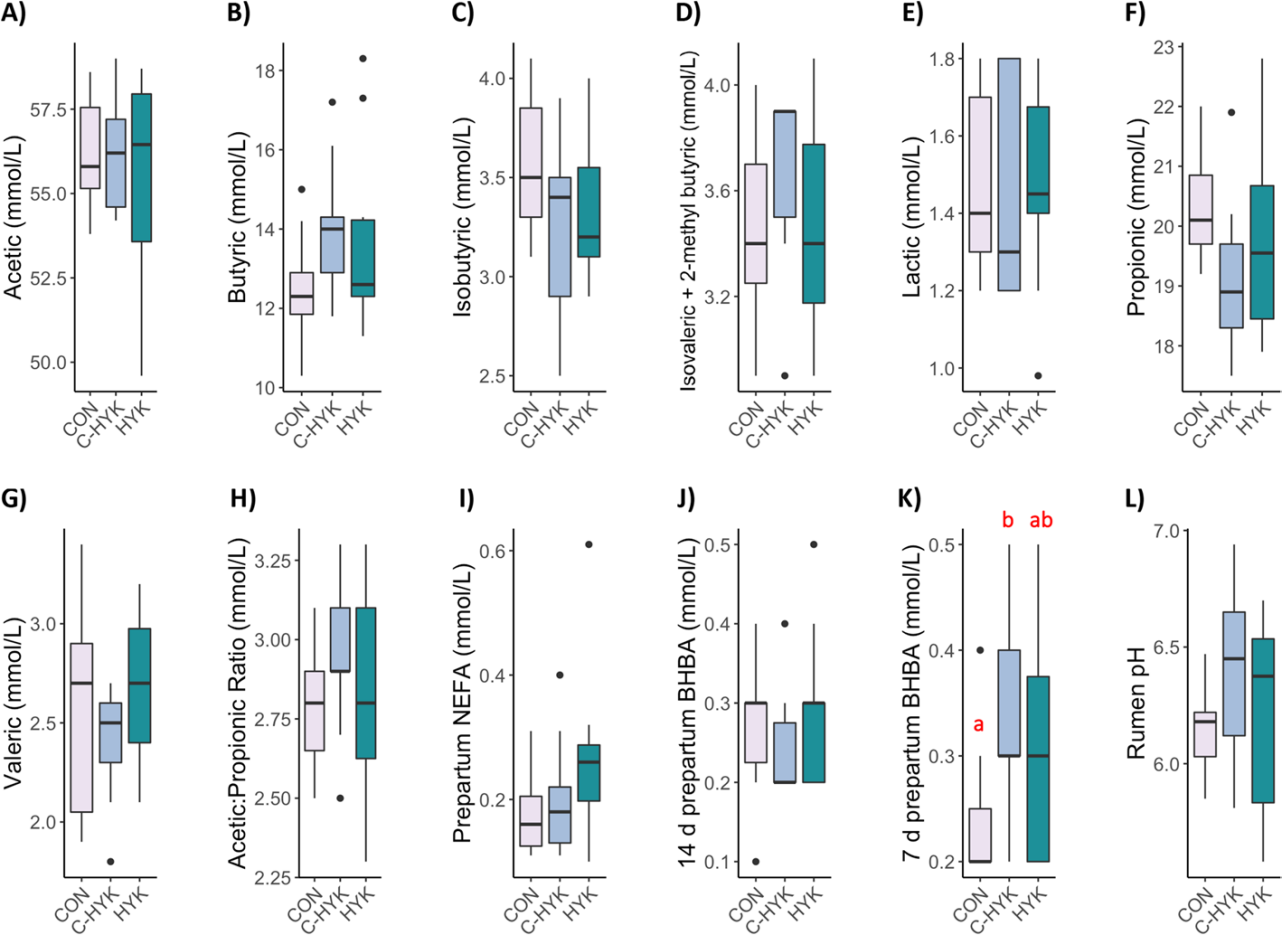
Distribution summaries of fatty acids and rumen pH. Boxplots describe the distribution of volatile fatty acids at time of rumenocentesis including **A**) acetic, **B**) butyric, **C**) isobutyric, **D**) isovaleric plus 2-methyl butyric, **E**) lactic, **F**) propionic, **G**) valeric, **H**) the ratio of acetic to propionic, as well as **I**) prepartum NEFA, **J**) 14 d prepartum BHBA, K) 7 d prepartum BHBA, and L) rumen pH at time of rumenocentesis. Distributions are stratified by health status category CON = control, C-HYK = control cows which later developed HYK, and HYK = hyperketonemic cows. Different lower-case letters represent groups which statistically significantly differ

Data distributions for metadata measures of interest (including all rumen fluid VFAs, prepartum blood NEFA, 14 d and 7 d prepartum blood BHB, and rumen pH) were stratified by health status: hyperketonemic cows (HYK, *n* = 10, classified based on a blood BHB concentration ^3^ 1.2 mmol/L within their first 14 DIM), control cows (CON, *n* = 11, cows were matched by parity and ± 3 DIM, with BHB < 1.2 mmol/L), and control-hyperketonemic cows (C-HYK, *n* = 9, cows originally enrolled as controls who later displayed BHB ^3^ 1.2 mmol/L after they were matched and were reclassified). These data were visualized via boxplots (Fig. 1). No statistically significant differences in values were observed among health groups for any measure, except 7 d prepartum BHB, in which C-HYK cows had higher BHB concentration than CON cows (*P* = 0.02). Body condition scores ranged from 2.75 to 3.75 and there was no statistically significant difference among health groups.

### Fecal microbiome

In sum, 592,769 fecal sequences passed quality control and were used in downstream analysis, with mean (± 1 standard deviation) 19,759 (± 2042) reads per sample. All measures of fecal alpha diversity including Shannon’s diversity index, Faith’s phylogenetic diversity, and Pielou’s evenness failed to distinguish CON, C-HYK, and HYK health status groups (Supplementary Fig. 1). Table 1 shows the correlation of these alpha diversity metrics to quantitative metadata. Shannon diversity appeared to be positively correlated with lactation number (r = 0.36, *P* = 0.05) and negatively correlated with prepartum NEFA measures (r = − 0.45, *P* = 0.01). Faith’s phylogenetic diversity varied by VFA concentration, such that fecal microbial diversity was negatively correlated with levels of the valeric acid (r = − 0.36, *P* = 0.05). In addition, Pielou’s evenness of fecal microbiota was negatively correlated with both prepartum NEFA levels (r − 0.43, *P* = 0.02) and BHB at time of rumenocentesis (r = − 0.39, *P* = 0.03).

**Table 1:** Summary of alpha diversity analyses. Pearson’s coefficients of correlation for Pielou’s evenness, Faith’s phylogenetic diversity, and Shannon diversity index with volatile fatty acids, beta-hydroxybutyrate (BHB) at various time points, prepartum non-esterified fatty acids (NEFA), rumen pH, body condition score, parity, and day in milk (DIM) of diagnosis or matching. Statistically significant correlations indicated by *P ≤ 0.05, **P ≤ 0.01

	FECAL			RUMEN		
	Pielou	Faith	Shannon	Pielou	Faith	Shannon
Acetic^a^	0.21	0.22	0.25	−0.35*	0.14	−0.03
Butyric^a^	−0.14	−0.17	− 0.07	0.29	0.04	0.09
Isobutyric^a^	−0.21	−0.17	− 0.27	−0.09	− 0.12	−0.17
Isovaleric plus 2-methyl butyric^a^	−0.16	0.08	−0.02	−0.19	0.33	0.14
Lactic^a^	−0.34	−0.01	− 0.31	0.25	− 0.06	−0.10
Propionic^a^	0.03	−0.11	−0.16	0.02	−0.37*	− 0.22
Valeric^a^	−0.17	− 0.36*	−0.30	0.13	−0.09	0.01
Ratio of Acetic to Propionic	0.10	0.16	0.22	−0.11	0.38*	0.20
Prepartum NEFA^a^	−0.43*	−0.26	− 0.45**	−0.05	− 0.20	−0.14
14 d prepartum BHB^a^	−0.14	−0.16	− 0.12	0.01	0.02	0.04
7 d prepartum BHB^a^	−0.02	−0.01	− 0.18	−0.10	0.14	0.00
BHB at diagnosis or matching^a^	−0.40*	−0.11	− 0.28	0.03	− 0.06	−0.05
DIM of diagnosis or matching	0.04	0.05	−0.08	−0.12	0.26	0.16
Rumen pH	0.09	0.20	0.11	−0.32	− 0.22	−0.37*
Body Condition Score	−0.02	−0.16	− 0.06	−0.07	0.005	0.00
Parity	0.23	0.27	0.36*	0.18	0.01	0.15

^a^mmol/L

**Fig. 2:**
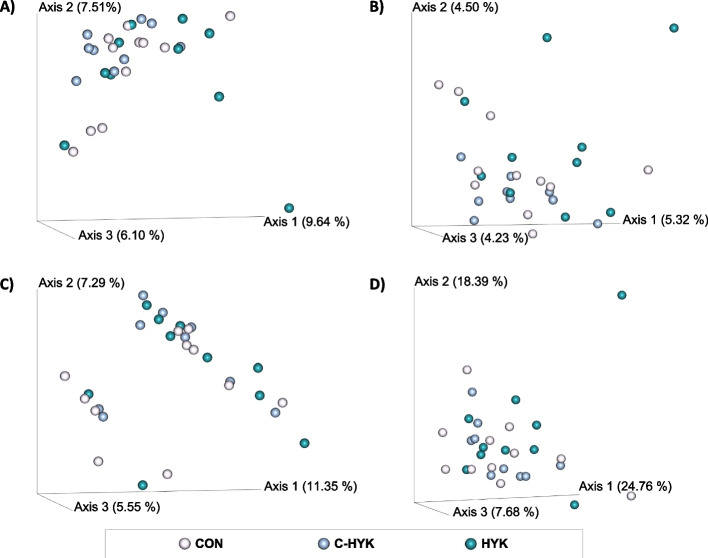
Fecal beta diversity metrics. The top 3 principal coordinates are plotted for each **A**) Bray-Curtis distances, **B**) Jaccard distances, **C**) unweighted Unifrac distances, **D**) weighted Unifrac distances. Each sphere represents an individual sample; samples are coded by health status group CON (pink), C-HYK (blue), and HYK (teal)

The top 3 principal coordinates (PC) from various beta diversity metrics are plotted in Fig. 2. Cows did not differentiate by health status according to Bray-Curtis, Jaccard, unweighted Unifrac, or weighted Unifrac distances calculated for the fecal microbiome (Fig. 2a-d). Unweighted Unifrac distance representation of the fecal microbial community identified a cluster of 9 cows that cannot be explained by any of our metadata (Fig. 2c). Both Bray-Curtis and Jaccard distances (Fig. 2a and b, respectively) reveal that fecal samples belonging to C-HYK cows are highly similar, though they are not distinguished overall from cows in other health groups. The C-HYK clusters can be identified by low PC1 and high PC2 Bray-Curtis scores (Fig. 2a), and low PC2 Jaccard scores (Fig. 2b).

**Fig. 3:**
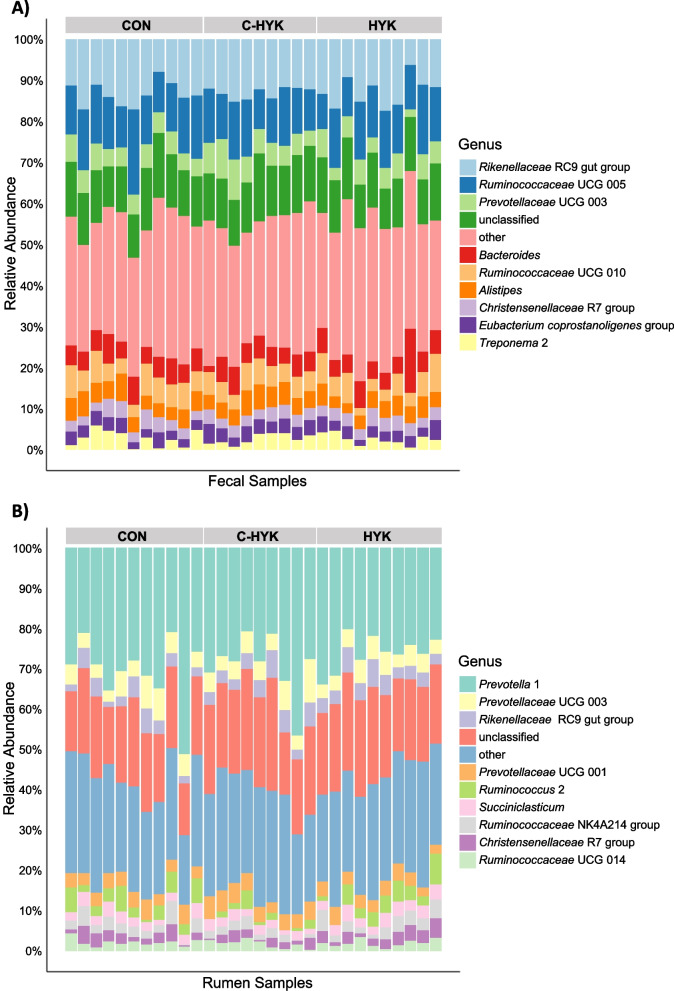
Taxonomic classification. The top 10 most abundant genera for each **A**) fecal and **B**) rumen samples are reported. Each column represents an individual sample (stratified by Health status CON = control, C-HYK = control-hyperketonemic, and HYK = hyperketonemic) and the relative abundance (%) of each genera is represented according to the colored legend

Less than 1% of fecal sample reads were unclassified at the phylum, class, and order level, 3.1% of reads were unclassified at the family level, 13.9% of reads unclassified at the genus level, and nearly all reads (99.4%) were unclassified at the species level. The most abundant genera in the feces were *Rikenellaceae* RC9 gut group, *Ruminococcaceae* UCG 005, *Prevotellaceae* UCG 003, *Bacteroides*, *Ruminococcaceae* UCG 010, *Alistipes*, *Christensenellaceae* R7, *Eubacterium coprostanoligenes* group, and *Treponema* 2 (Fig. 3a). No major differences in taxonomy were observed among health status groups.

The most appropriate multinomial regression model to compute log-fold changes in fecal microbial abundance relative to health status included parity as a fixed effect. Convergence summaries evaluating both the given model and the baseline null model can be found in Supplementary Fig. 2. The *Q*_2_ score comparing these models was − 0.18.

### Rumen microbiome

A total of 2,340,585 rumen sequences passed quality control and averaged 75,503 (± 22,431) reads per sample. All measures of rumen alpha diversity including relative abundance, Shannon’s diversity index, Faith’s phylogenetic diversity, and Pielou’s evenness failed to distinguish CON, C-HYK, and HYK health status groups (Supplementary Fig. 1). Table 1 shows the correlation of these alpha diversity metrics to quantitative metadata. Shannon diversity was negatively correlated with rumen pH at time of rumenocentesis (r = − 0.37, *P* = 0.04). Faith’s phylogenetic diversity varied by VFA concentration among rumen microbial populations; rumen microbial diversity was negatively correlated with propionic acid (r = − 0.37, *P* = 0.04) but positively correlated with the ratio of acetic to propionic acid (r = 0.38, *P* = 0.04). Among rumen samples, Pielou’s evenness was negatively correlated with the VFA acetic acid concentration (r = − 0.35, *P* = 0.05).

**Fig. 4:**
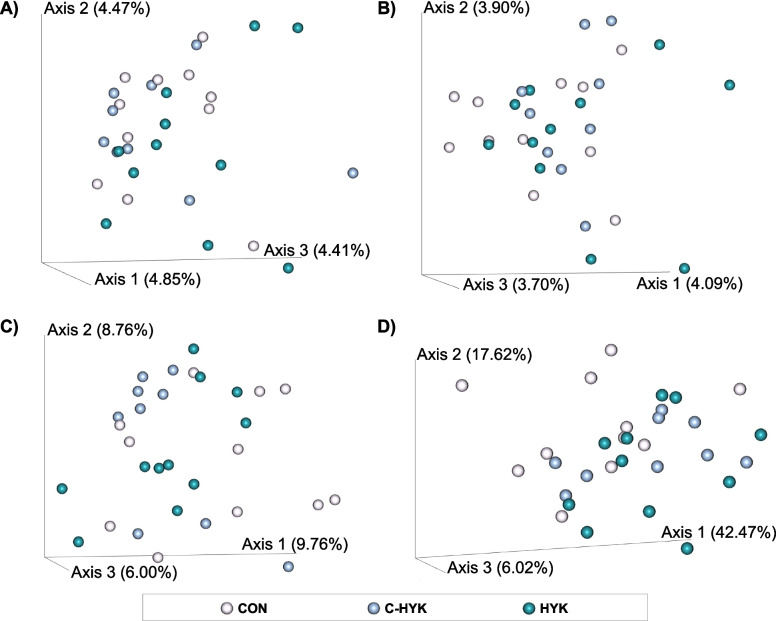
Rumen beta diversity metrics. The top 3 principal coordinates are plotted for each **A**) Bray-Curtis distances, **B**) Jaccard distances, **C**) unweighted Unifrac distances, **D**) weighted Unifrac distances. Each sphere represents an individual sample; samples are coded by health status group CON (pink), C-HYK (blue), and HYK (teal)

The top 3 principal coordinates from various beta diversity metrics are plotted in Fig. 4. Cows did not differentiate by health status according to Bray-Curtis, Jaccard, or unweighted Unifrac distances calculated for the rumen microbiome (Fig. 4a-c). Pairwise PERMANOVA testing revealed a statistically significant difference in weighted Unifrac distances among CON and HYK rumen microbial communities (*P* = 0.03; Fig. 4d). The C-HYK cows tended to cluster together for all beta diversity metrics.

Less than 1% of rumen sample reads were unclassified at the phylum, class, and order level, 7.6% of reads were unclassified at the family level, 20.2% of reads unclassified at the genus level, and nearly all reads (92.6%) were unclassified at the species level. The most abundant genera in the rumen were *Prevotella* 1, *Prevotellaceae* UCG 003, *Rikenellaceae* RC9 gut group, *Prevotellaceae* UCG 001, *Ruminococcus* 2, *Succiniclasticum*, *Ruminococcaceae* NK4A214 group, *Christensenellaceae* R7 group, and *Ruminococcaceae* UCG 014 (Fig. 3b). C-HYK cows had a significantly lower abundance of *Ruminococcus* 2 compared to both HYK and CON cows (*P*_*adj*_ < 0.05), but no other differences in taxonomy were observed among health status groups.

The most appropriate multinomial regression model to compute log-fold changes in rumen microbial abundance relative to health status included rumen pH as a fixed effect. Convergence summaries evaluating both the given model and the baseline null model can be found in Supplementary Fig. 3. The *Q*_2_ score comparing these models was − 0.07.

## Discussion

It has been well-established that hyperketonemic cows may experience elevated prepartum serum BHB levels, which corroborates the observed increase in 7 d prepartum BHB in C-HYK cows compared to CON cows (Fig. 2k, *P* = 0.02), and suggests these measures could have predicted the eventual HYK diagnosis of the C-HYK cows originally enrolled as matched controls [12]. Interestingly, HYK cows displayed a broad range of 7 d prepartum BHB concentrations which overlapped with both CON and C-HYK cow values, implying this measure alone is not sufficient to indicate the onset of metabolic disease.

### Fecal microbiome

The positive correlation between the Shannon diversity index and lactation number (r = 0.36, *P* = 0.05) suggests that older cows have greater fecal microbial abundance and distribution, which may be due to their increased exposure time to the environment. Similar age-related changes in alpha diversity measures have been observed in humans, with the eventual plateau and decline of diversity as individuals near the end of their life expectancy [13, 14]. However, several surveys of dairy farms have not found parity to be significantly correlated with bovine fecal alpha diversity, and this disparity may be explained by farm-specific influences on the microbiome, such as management and diet [15, 16]. Shannon diversity was also negatively correlated with 7 d prepartum NEFA measures (r = − 0.45, *P* = 0.01); similarly, Pielou’s evenness of fecal microbiota was negatively correlated with both 7 d prepartum NEFA levels (r − 0.43, *P* = 0.02) and BHB at time of rumenocentesis (r = − 0.39, *P* = 0.03). Serum concentrations of both NEFA and BHB have been well-established as predictors of clinical disease during the transition period, which supports the inverse relationship we observed between fecal alpha diversity and prepartum NEFA and BHB levels [17].

The clustering of C-HYK cows revealed in our ordination analyses suggests that the fecal microbiomes of these cows are highly similar, though they do not segregate from either HYK or CON cows. Both Bray-Curtis and Jaccard ordination (Fig. 2a and b, respectively) identify a tight grouping of C-HYK fecal microbiomes, whereas greater distances are evident among CON and HYK fecal microbial communities. This suggests that while the fecal microbiome does not distinguish healthy and hyperketonemic cows, the fecal microbial profile of cows which show a later onset of hyperketonemia in early lactation is unique. Because C-HYK cows were sampled at time of matching not at time of onset of HYK, perhaps their unique microbial profiles can be considered an indicator of future metabolic distress.

Genera including *Rikenellaceae* RC9 gut group, *Bacteroides*, and *Alistipes*, which were among the most represented taxa in our data, have been strongly associated with the fecal microbiome [18]. Various *Eubacterium* genera have been discovered to be enriched in beef cattle feces [19]. Interestingly, we found *Prevotellaceae* and *Ruminococcaceae* genera were relatively highly abundant in the fecal microbiome, whereas they have been previously reported as core members of the rumen microbiota [19–21]. Similarly, *Christensenellaceae* genera have been previously linked to both fecal and rumen microbiota, and *Treponema* genera have been associated with the rumen microbiota, though at relatively lower abundance [21–23]. Taxonomic classification revealed fecal microbial features we expected to be present in the bovine gastrointestinal tract. However, no major differences were observed in most abundant taxa among health status groups, suggesting that hyperketonemia is not influenced by fecal microbial composition, and vice versa.

The evaluation of the fit of our most appropriate regression to compute log-fold changes in fecal microbial abundance relative to health status revealed that our model did not have predictive value (*Q*_2_ < 0). In addition, the high cross validation scores suggest that with these data we cannot accurately predict microbial features with respect to health status, and the null model comparison suggests that including our metadata actually decreased the estimated predictive accuracy (Supplementary Fig. 2a). Similarly, we were not able to reduce the error of the training samples across iterations, indicating the model does not fit our data well (Supplementary Fig. 2b). Consequently, we were not able to employ compositional methods to gain insight on the role of certain microbial features in health status. This is likely due to insufficient power, an artifact of the small sample size of our different health statuses which were a consequence of the unexpected development of hyperketonemia in a subset of the controls (C-HYK group).

No other studies have related the bovine fecal microbiome to hyperketonemia.

### Rumen microbiome

The negative correlation between the Shannon diversity index and rumen pH (r = − 0.37, *P* = 0.04) suggests that while all cows were within the normal rumen pH range of 5.5 to 7, cows with higher rumen pH at time of sampling tended to have lower abundance and evenness of rumen microbes [24]. Rumen pH tended to be higher among C-HYK and HYK cows compared to CON cows, though there was no statistically significant difference among groups. Rumen pH can vary over the course of a day depending on intake patterns, but this study does not have the necessary data to investigate the hypothesis that HYK and C-HYK cows exhibit different feeding behaviors than CON cows. Various alpha diversity metrics varied by VFA concentration; Faith’s phylogenetic diversity was negatively correlated with propionic acid (r = − 0.37, *P* = 0.04) but positively correlated with the ratio of acetic to propionic acid (r = 0.38, *P* = 0.04), Pielou’s evenness was negatively correlated with the VFA acetic acid concentration (r = − 0.35, *P* = 0.05). One possible explanation for these observations is that cows with higher concentrations of propionic or acetic acid in the rumen may have a commensal population skewed towards the microbes responsible for the production of VFAs, though neither *Propionibacterium* nor *Acetobacter* genera were observed in high abundance in our data. Increased bovine rumen fermentation rate has been previously associated with a decrease in microbial species diversity, affirming these observations [25].

As observed in the fecal microbiome analysis, C-HYK cows tended to cluster together for all beta diversity metrics, whereas HYK and CON rumen microbial populations appeared more dissimilar among samples (Fig. 4). The statistically significant difference in weighted Unifrac distances among CON and HYK rumen microbial communities (*P* = 0.03; Fig. 4d) suggests that hyperketonemia may be reflected in rumen microbial community composition, or vice versa. In this study design we are unable to determine whether the development of hyperketonemia causes dysbiosis, or whether perturbances in the microbiome occur and subsequently induce metabolic disease.

The most abundant taxa in the rumen included *Prevotella*, *Prevotellaceae*, *Rikenellaceae*, *Ruminococcus*, *Ruminococcaceae*, *Succiniclasticum*, and *Christensenellaceae* genera, all of which have been previously associated with the bovine rumen microbiota [18]. In particular, *Prevotella* genera have been associated as core members of the rumen microbiota, and they have been implicated in the production of both acetate and propionate [26]. The high abundance of *Prevotella* may also explain the inverse relationship between alpha diversity and acetic and propionic acid concentrations; the rumen microbial composition appears skewed towards a taxon which produce those VFAs. The lower abundance of *Ruminococcus* 2 in C-HYK compared to both HYK and CON cows (*P*_*adj*_ < 0.05) suggests cows with late onset of hyperketonemia may be less efficient at degrading cellulose, though we do not yet have a complete understanding of the mechanistic role of *Ruminococcus* spp. in rumen metabolism [27]. A recent study found high covariance between rumen microbial composition and milk BHB in Danish Holsteins but did not focus on early lactation cows, so were not able to provide microbiome insights during the transition period [28]. These researchers found that *Ruminococcaceae* families were reduced as concentrations of milk BHB increased, in theme with our findings that C-HYK cows experienced lower abundance of the genus *Ruminococcus* 2. *Ruminoccocus* sp. are known to digest a variety of fibers and produce acetate; however, we did not observe lower acetate production in C-HYK cows (Fig. 1), suggesting that their relatively lower abundance of *Ruminococcus* 2 did not greatly impact VFA production and usable energy. However, because no other differences were observed among health status groups, it appears that hyperketonemia is not greatly influenced by the most abundant rumen taxa, and vice versa.

The evaluation of the fit of our most appropriate regression to compute log-fold changes in rumen microbial abundance relative to health status revealed that our model did not have predictive value (*Q*_2_ < 0). High cross validation scores suggest that with these data we cannot accurately predict rumen microbial features with respect to health status, and because our regression failed to outperform the null model, it seems including our metadata actually decreased the estimated predictive accuracy (Supplementary Fig. 3a). Similarly, we were not able to reduce training sample errors across iterations, though our regression performed slightly better than the null, indicating the model did not fit our data well (Supplementary Fig. 3b). Consequently, we were not able to employ compositional methods to gain insight on the role of certain rumen microbial features in health status. Again, this is likely due to insufficient power resulting from the small sample sizes of our three different health groups.

Microbiome studies in animal sciences most commonly employ 16S rRNA sequencing methods due to its relative affordability and the increasing availability of powerful and user-friendly bioinformatic tools [29]. However, a limitation of 16S rRNA sequencing is that it cannot describe the activity of a microbial community, their metabolic potential, or even whether the microbes are alive or dead. This study provides an initial survey of microbial communities in cases of hyperketonemia or health, but greater insights into potential mechanisms at play would require alternative -omics approaches, such as shotgun sequencing, meta-transcriptomics, or meta-proteomics [30].

## Conclusions

The aims of this study were to 1) determine whether the development of hyperketonemia in early lactation dairy cows is indicated by their gut microbiome, and 2) to identify microbial features which may inform health status. No differences in fecal or rumen alpha diversity or taxonomic composition were observed among health groups, except for lower relative abundance of *Ruminococcus* 2 in the rumen of C-HYK cows, which suggests these cows may be less efficient at degrading cellulose, though we do not yet have a complete understanding of the mechanistic role of *Ruminococcus* spp. in rumen metabolism. Cows did not differentiate by health status according to fecal microbial beta diversity metrics, though pairwise PERMANOVA testing revealed statistically significant differences in weight Unifrac distances between CON and HYK rumen microbial communities. Short distances were observed among C-HYK cow fecal and rumen microbiomes, suggesting that cows which initially appear health with late onset of HYK were highly similar. Multinomial regressions used to compute log-fold changes in microbial abundance relative to health status were not found to have predictive value. Substantial differences in fecal or rumen microbiomes among cows experiencing different levels of negative energy balance was not indicated, suggesting that HYK may not be influenced by gut microbial composition, and vice versa.

## Methods

### Study population

Data were collected from a single commercial dairy farm in Northern Colorado from May to August 2014. The farm milked approximately 1200 Holstein cows three times a day with an average daily milk production of 35.8 kg with 3.7% fat and 3.2% protein per cow and a bulk tank somatic cell count of 170,000 cells/mL throughout the study period. Nulliparous cows were kept in a separate dry lot pen from primiparous and multiparous cows for the 60 days before expected calving and comingled in a different dry lot pen when parturition was imminent. All cows were then moved to a single free-stall pen kept at an 85% stocking density for the first 30 d in milk (DIM). Both pre-fresh and fresh cows were fed ad libitum total mixed rations.

### Study design and data collection

**Fig. 5:**
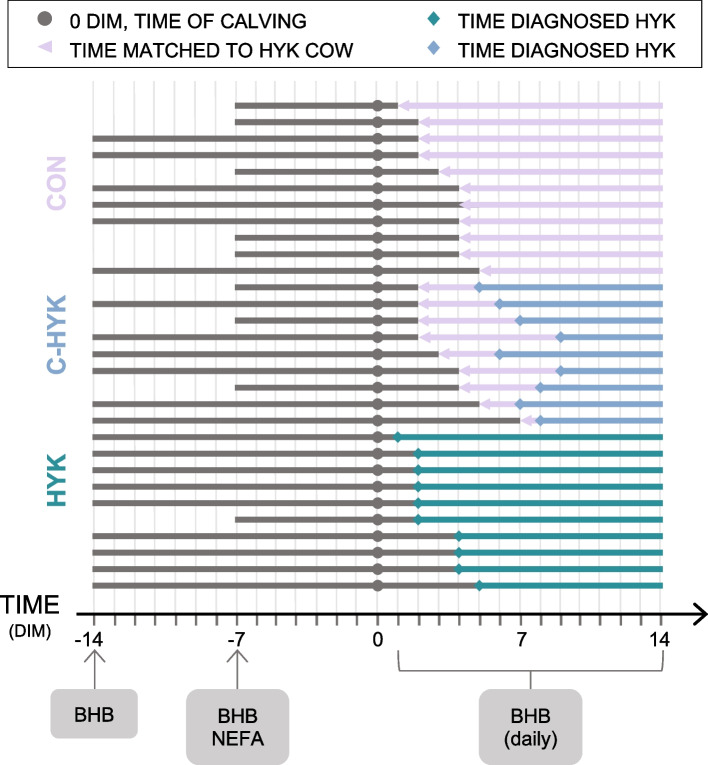
Study design schematic. Cows (*N* = 30) were enrolled at 14 or 7 days prepartum (DPP) based on their expected calving date and their blood drawn for beta-hydroxybutyrate (BHB) analysis. Non-esterified fatty acids (NEFA) were also measured at 14 DPP. After calving (gray circle), blood BHB was measured daily for 14 d to determine hyperketonemia (HYK, *n* = 10) status. Pink triangles indicate the d in milk (DIM) healthy cows (*n* = 20) were matched by parity and DIM with HYK cows. Teal diamonds indicate DIM of HYK diagnosis; blue diamonds indicate DIM of HYK diagnosis for cows initially believed to be healthy (C-HYK, *n* = 9)

We conducted a prospective nested case-control study in which cows were enrolled 14 to 7 d before expected calving and followed through their first 14 DIM (Fig. 5). Blood samples were collected at 14 and 7 d prepartum (DPP) from the coccygeal vessels using a 20-gauge, 2.54 cm needle and blood collection tube without anticoagulant for analysis of NEFA and β-hydroxybutyrate (BHB) concentrations. A Precision Xtra meter (Abbott Laboratories, Abbott Park, IL) was used cow-side to determine BHB concentrations. The remainder of the blood was allowed to clot for 45 minutes at room temperature and transported at approximately 4 °C to Colorado State University for more processing. Samples were centrifuged within 2 hours of collection for 10 minutes at 2000 x *g* and 20 °C; serum was harvested and frozen at − 80 °C until NEFA analysis. Postpartum blood samples were collected in the same manner and BHB concentrations determined daily from 1 to 14 DIM.

Hyperketonemic cows (HYK, *n* = 10) were classified based on a blood BHB concentration ^3^ 1.2 mmol/L within their first 14 DIM. For each HYK cow, two non-hyperketonemic (CON, *n* = 20) cows were matched by parity and ± 3 DIM, with BHB < 1.2 mmol/L. Daily blood BHB measures were used to confirm CON cows maintained their healthy status, however, it was observed that some CON cows displayed BHB ^3^ 1.2 mmol/L after they were matched, and these cows were reclassified as control-hyperketonemic (C-HYK, *n* = 9). At time of HYK diagnosis or CON matching, all cows underwent one rumenocentesis performed by a single, trained veterinarian according to the following protocol. A 5 cm square area was clipped 5 cm caudal to the left 13th rib at a horizontal level 5 cm dorsal to the top of the olecranon. The area was surgically prepared 3 times using gauze soaked in 10% povidone-iodine followed by 70% isopropyl alcohol, and a local block of the skin and muscle layers with 5 mL of 2% lidocaine. A rumenocentesis needle was placed straight into or up to a 35° cranial angle to the horizontal plane into the rumen, and approximately 20 mL of rumen fluid was evacuated into a sterile 35 mL syringe case. Rumen fluid samples were immediately evaluated for pH using a Horiba LAQUA Twin pH meter (Hitachi, Ltd., Tokyo, Japan). The remainder of each rumen fluid samples was divided into two equal portions for volatile fatty acid (VFA) and microbiome analyses. The VFA portion had 1 mL of 25% meta-phosphoric acid added, and both portions were snap-frozen in liquid nitrogen on farm and then transported to the laboratory where they were stored at − 80 °C until analysis. A fecal sample was also obtained from each cow on the day of rumenocentesis. Body condition was scored for each cow on the day of rumenocentesis by a single, trained veterinarian on a scale of 1 to 5 in 0.25 increments [31].

After CON, C-HYK, and HYK classification, prepartum blood serum samples were submitted to the New York State Animal Health Diagnostic Center for NEFA concentration determination on a Roche Modular P chemistry analyzer (Roche, Basel, Switzerland). Concentrations of VFAs from rumen fluid (acetic, butyric, isobutyric, isovaleric plus 2-methyl butyric, lactic, propionic, and valeric acids) were determined via gas chromatography as described by Erwin et al. (1961) at the Department of Animal Sciences, Cornell University [32].

### DNA extraction, 16S rRNA amplification, and sequencing

Rumen fluid and fecal samples were homogenized, and 250 mg aliquots were processed using MO BIO PowerSoil DNA Isolation Kit (Qiagen, Inc., Germantown, MD) according to the manufacturer protocol. For microbiome analysis, V4 hypervariable region of the 16S rRNA gene was amplified following the Earth Microbiome Project (https://earthmicrobiome.org) protocol [33, 34]. Amplicons were then sequenced in an Illumina MiSeq platform (Illumina, Inc., San Diego, CA) using a single-indexing approach at the Department of Population Medicine and Diagnostic Sciences, Cornell University.

The V4 hypervariable region of the bacterial 16S rRNA gene was amplified using primers 515F 5′ GTGCCAGCMGCCGCGGTAA 3′ and uniquely 5′ barcoded 806R 5′ GGACTACHVGGGTWTCTAAT 3′ [3]. Amplicons were generated in triplicate with 2X EconoTaq® Plus Green Master Mix (Lucigen, Middleton, WI), including 12–300 ng of extracted DNA and 10 μM of each primer. Replicate amplicons were pooled, verified via gel electrophoresis on 1.2% agarose gels stained with 0.5 mg/mL ethidium bromide, and purified with a QIAquick PCR Purification Kit (Qiagen, Inc., Germantown, MD). Positive and negative controls were processed in parallel. Purified amplicons were quantified via Quant-iTä PicoGreenä dsDNA Assay (Invitrogen, Carlsbad, CA). Aliquots of all amplicons were standardized to the same concentration and separate equimolar libraries constructed for each rumen and fecal samples. These libraries were sequenced in two separate runs of the MiSeq platform (Illumina, Inc., San Diego, CA) using reagent kit v2 (300 cycles).

### Statistical and metagenomic analyses

All code and data are publicly available at https://github.com/gandalab/HYK-gut-microbiome. Metadata measures of interest (including all VFAs, prepartum NEFA, 14 d and 7 d prepartum BHB, and rumen pH) were stratified by health status HYK (*n* = 10), C-HYK (*n* = 9), and CON (*n* = 11) and normality checked via Shapiro-Wilk test [35]. Data distributions were visualized via boxplots and statistically significant differences in values among health status groups tested via non-parametric Kruskal-Wallis rank rum tests at α < 0.05 [36]. In the case a statistically significant difference was indicated by the Kruskal-Wallis test, a Dunn test with false discovery rate multiple testing correction was run post-hoc to determine which specific categorical levels differed [37]. All statistical analyses of metadata were performed in R version 3.6.2 (2019-12-12) [38].

Rumen and fecal microbiomes were sequenced in different batches and therefore analyzed separately with no direct comparison, and bioinformatics were performed using QIIME 22020.6 [39]. Single-end raw 16S rRNA sequences were de-multiplexed and quality filtered, and then denoised via the q2-dada2 plugin [40]. All sequences were aligned using the mafft program from the q2-phylogeny plugin and then FastTree applied to create a phylogenetic tree, rooted at the midpoint of the longest tip-to-tip distance [41, 42]. Rumen and fecal samples were rarefied to 17,429 and 13,237 sequences per sample, respectively. Subsequently, alpha and beta diversity metrics were calculated including observed features, Faith’s phylogenetic diversity, Shannon’s diversity index, Pielou’s evenness, Jaccard distances, Bray-Curtis distances, and unweighted and weighted Unifrac distances [43–46]. The q2-feature-classifier plugin was used to assign taxonomy via fit classifier naïve Bayes against the Silva_132_release 99% 16S reference sequences [47, 48]. This workflow resulted in classification of reads at the taxonomic levels of kingdom, phylum, class, order, family, genus, and species. A Kruskal-Wallis and post-hoc Dunn test with false discovery rate multiple testing correction was used to identify significant differences in relative abundance among the three health status groups [36, 37]. Pearson’s correlation and Kruskal-Wallis tests were used to determine statistically significant differences in alpha diversity by metadata measures of interest. Permutational multivariate analysis of variance (PERMANOVA) was used to assess statistically significant differences in beta diversity among samples [49].

To mitigate the bias constant introduced by unknown microbial load per sample, we utilized compositional approaches to associate key microbes with health status [50]. Using the QIIME 2 plug-in “Songbird”, we performed a multinomial regression whose primary output was differentials describing the log-fold change of features with respect to health status [51]. All models were trained and tested on randomly assigned samples balanced across all 3 health status groups. All relevant covariates were considered, including parity, rumen pH, VFA concentration, and prepartum NEFA and BHB. Model fit was assessed by evaluating convergence summaries and comparing to a null model to confirm our regressions had predictive value. A *Q*^2^ score was used to quantify the performance of each model compared to the null and is given by ${Q}^2=1-\frac{m_1}{m_2}$, where *m*_1_ indicates the average absolute model error, and *m*_2_ indicates the average absolute null model error (as described in: https://github.com/biocore/songbird). Output differentials were ranked and visualized via Qurro (Quantitative Rank/Ratio Observations) to identify the top 5% of microbial features associated with each CON, C-HYK, and HYK health status [52].

## Supplementary Information

**Additional file 1: Supplementary Fig. 1.** Alpha diversity metrics by health status group. Boxplots showing the distribution of fecal and rumen alpha diversity for each health group control (CON, *n* = 11), control-hyperketonemic (C-HYK, *n* = 9), and hyperketonemic (HYK, *n* = 10), as measured by Pielou’s evenness, Faith’s phylogenetic diversity, and the Shannon diversity index.

**Additional file 2: Supplementary Fig. 2.** Fecal microbiome convergence summaries. The A) cross-validation score and B) loss plots are shown for the Songbird multinomial regression model used to compute log-fold changes in fecal microbial abundance relative to health status. The blue line represents our model computing log-fold changes with respect to health status; the orange line represents the null or baseline model demonstrating log-fold changes due to random chance.

**Additional file 3: Supplementary Fig. 3.** Rumen microbiome convergence summaries. The A) cross-validation score and B) loss plots are shown for the Songbird multinomial regression model used to compute log-fold changes in rumen microbial abundance relative to health status. The blue line represents our model computing log-fold changes with respect to health status; the orange line represents the null or baseline model demonstrating log-fold changes due to random chance.

## Data Availability

The datasets used and/or analyzed during the current study are available from the corresponding author on reasonable request.

## Abbreviations

NEFA: Non-Esterified Fatty Acids
DIM: Days In Milk
DPP: Days Pre-Partum
BHB: Beta-Hydroxybutyrate
HYK: Hyperketonemic
CON: Control
C-HYK: Control-Hyperketonemic
VFA: Volatile Fatty Acids
PERMANOVA: Permutational Multivariate Analysis of Variance
PC: Principal Coordinate
