# Supplementary figures and images for: The association of hyperketonemia with fecal and rumen microbiota at time of diagnosis in a case-control cohort of early lactation cows
Source: BMC Vet Res. 2022 Nov 21;18:411. doi: 10.1186/s12917-022-03500-4 (PMC9677665; doi:10.1186/s12917-022-03500-4)

Pielou's Evenness

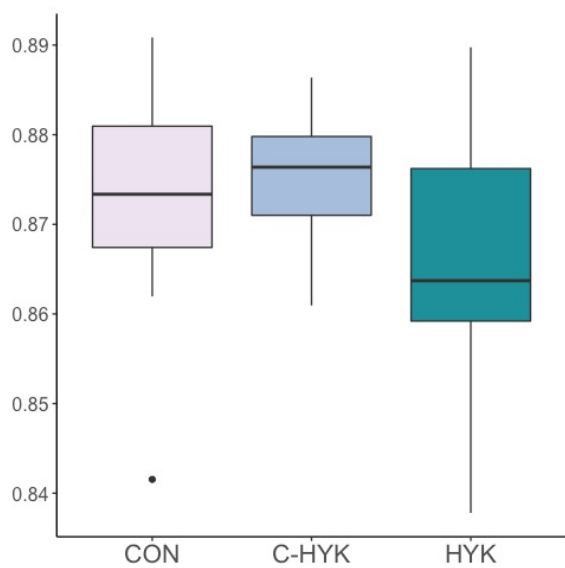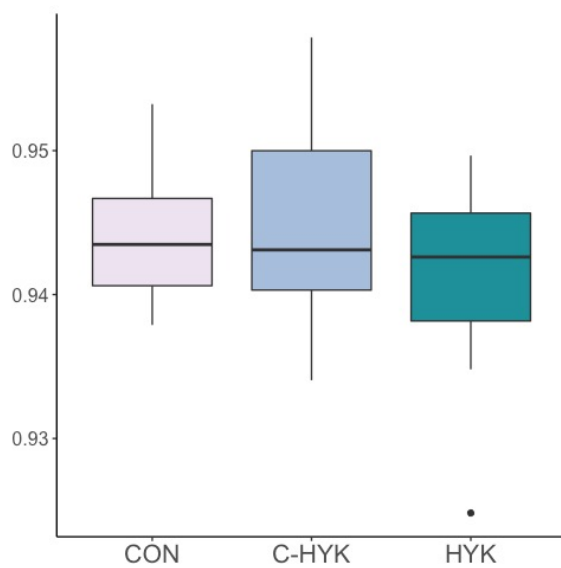

Faith's Phylogenetic Diversity

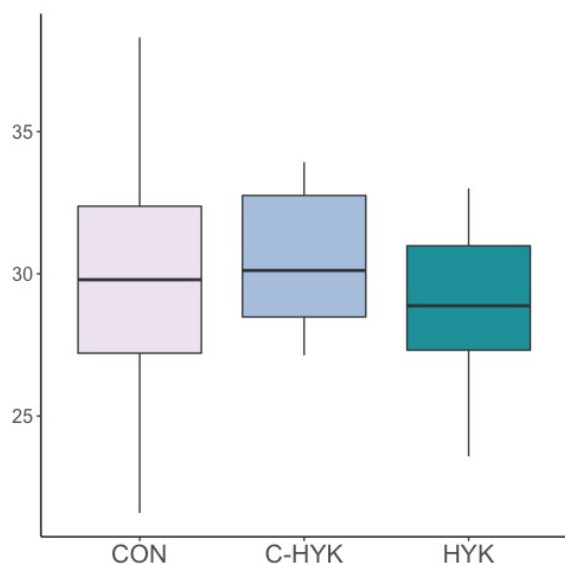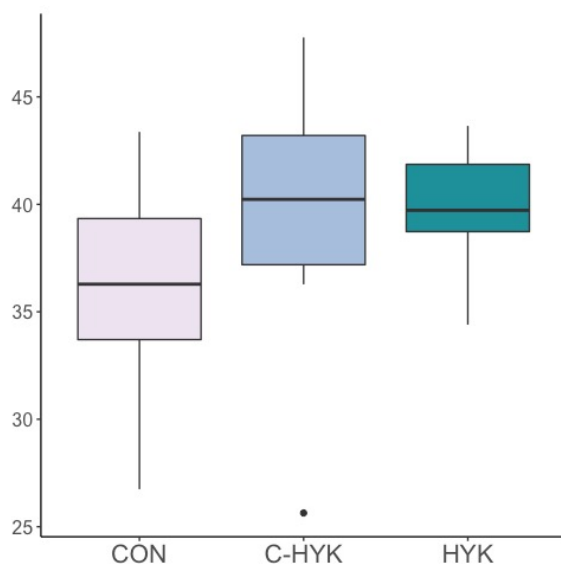

Shannon Diversity

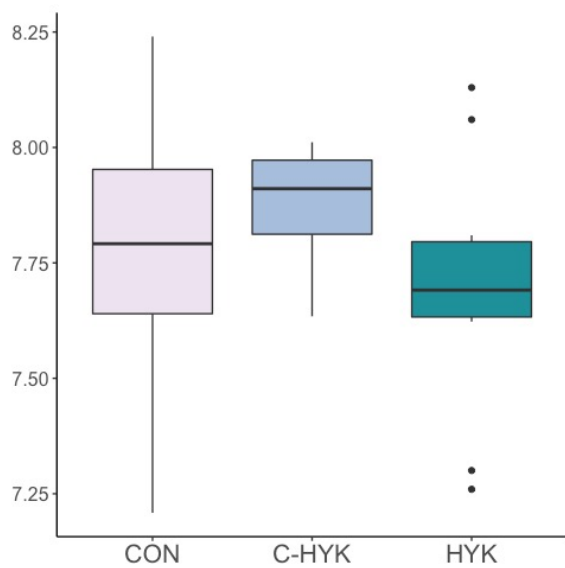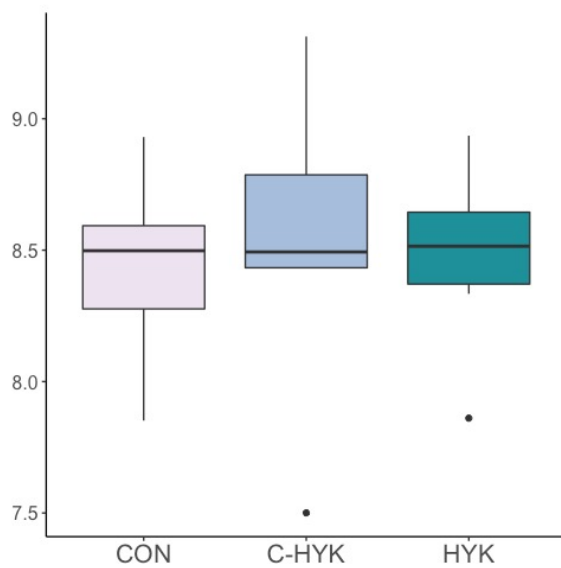

Supplement: Supplementary file 1 — Additional file 1: Supplementary Fig. 1. Alpha diversity metrics by health status group. Boxplots showing the distribution of fecal and rumen alpha diversity for each health group control (CON, n = 11), control-hyperketonemic (C-HYK, n = 9), and hyperketonemic (HYK, n = 10), as measured by Pielou’s evenness, Faith’s phylogenetic diversity, and the Shannon diversity index. [file 12917_2022_3500_MOESM1_ESM.pdf]

A

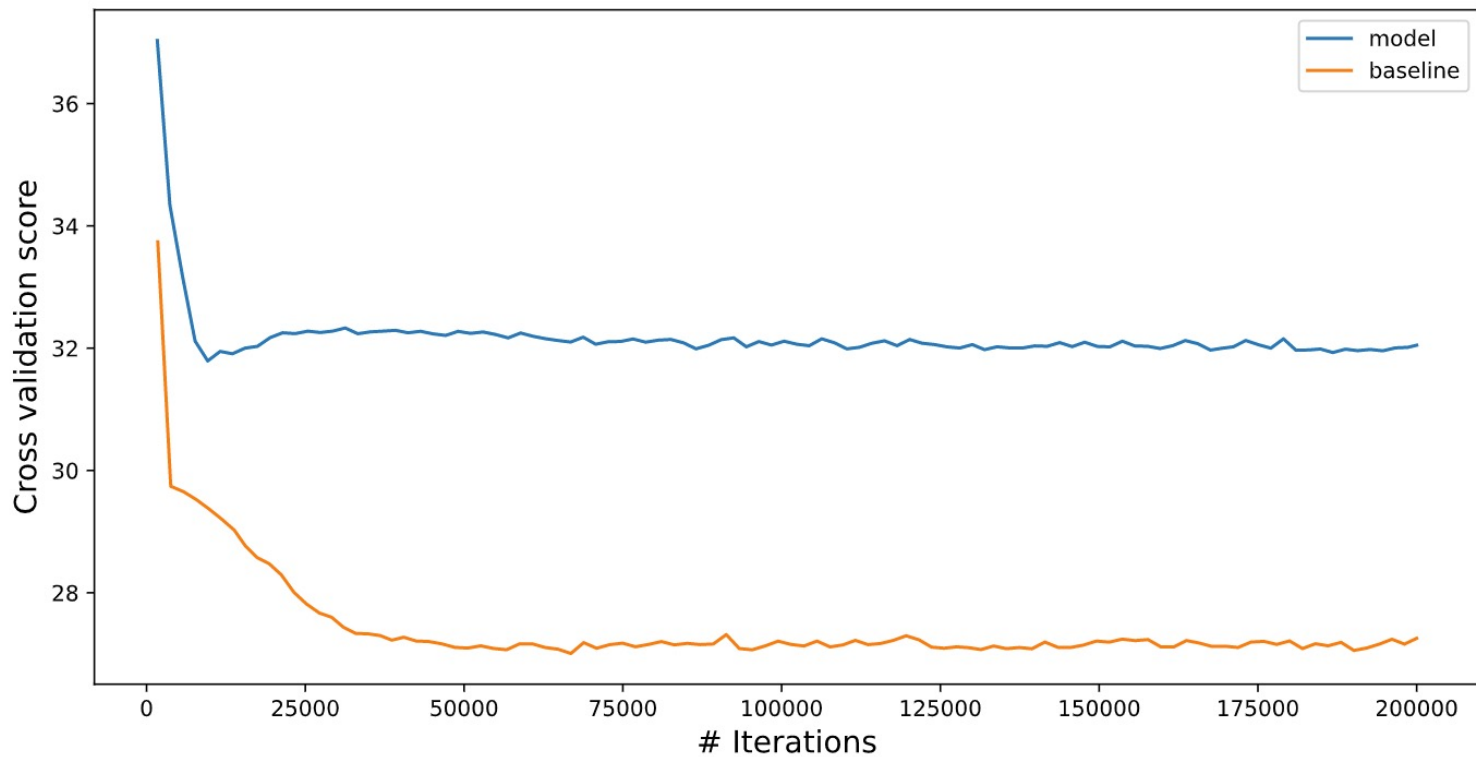

B

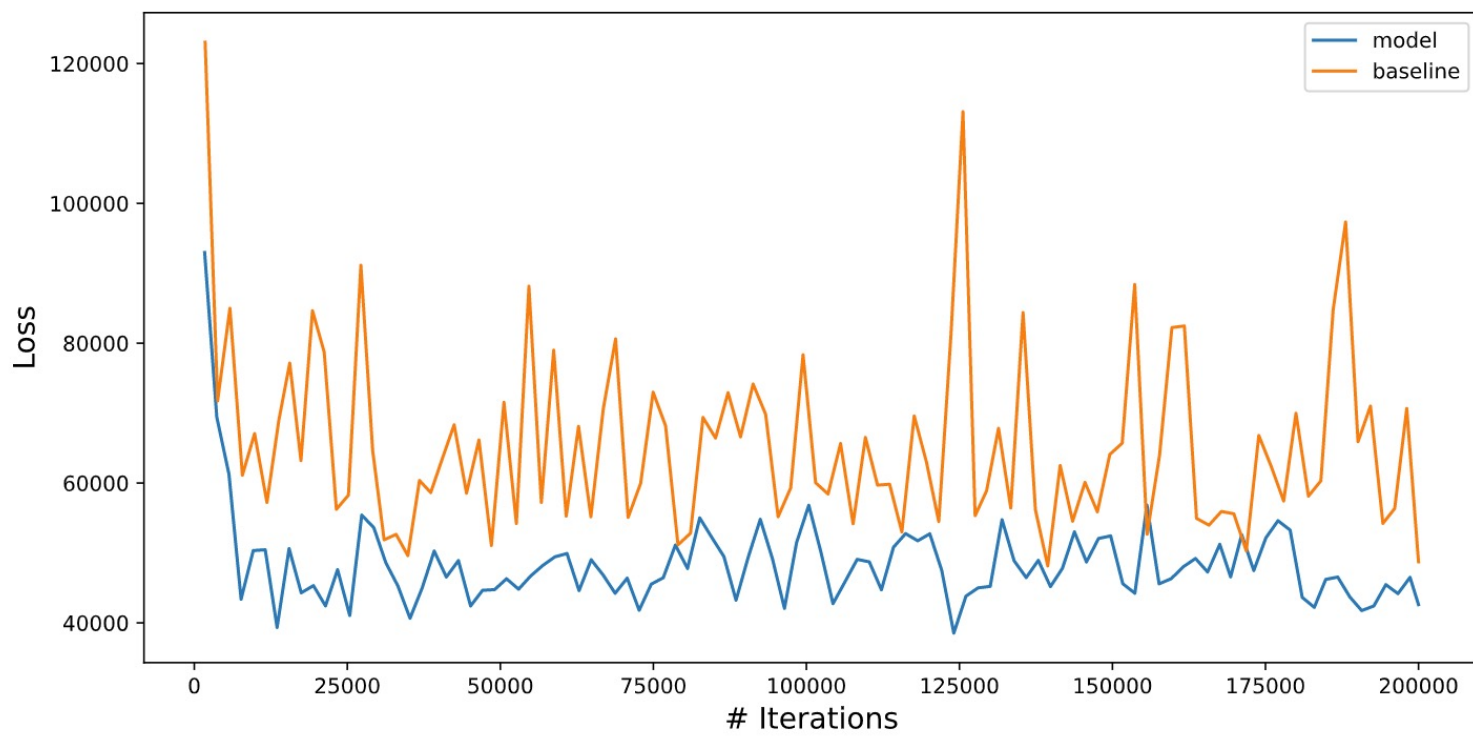

Supplement: Supplementary file 2 — Additional file 2: Supplementary Fig. 2. Fecal microbiome convergence summaries. The A) cross-validation score and B) loss plots are shown for the Songbird multinomial regression model used to compute log-fold changes in fecal microbial abundance relative to health status. The blue line represents our model computing log-fold changes with respect to health status; the orange line represents the null or baseline model demonstrating log-fold changes due to random chance. [file 12917_2022_3500_MOESM2_ESM.pdf]

A

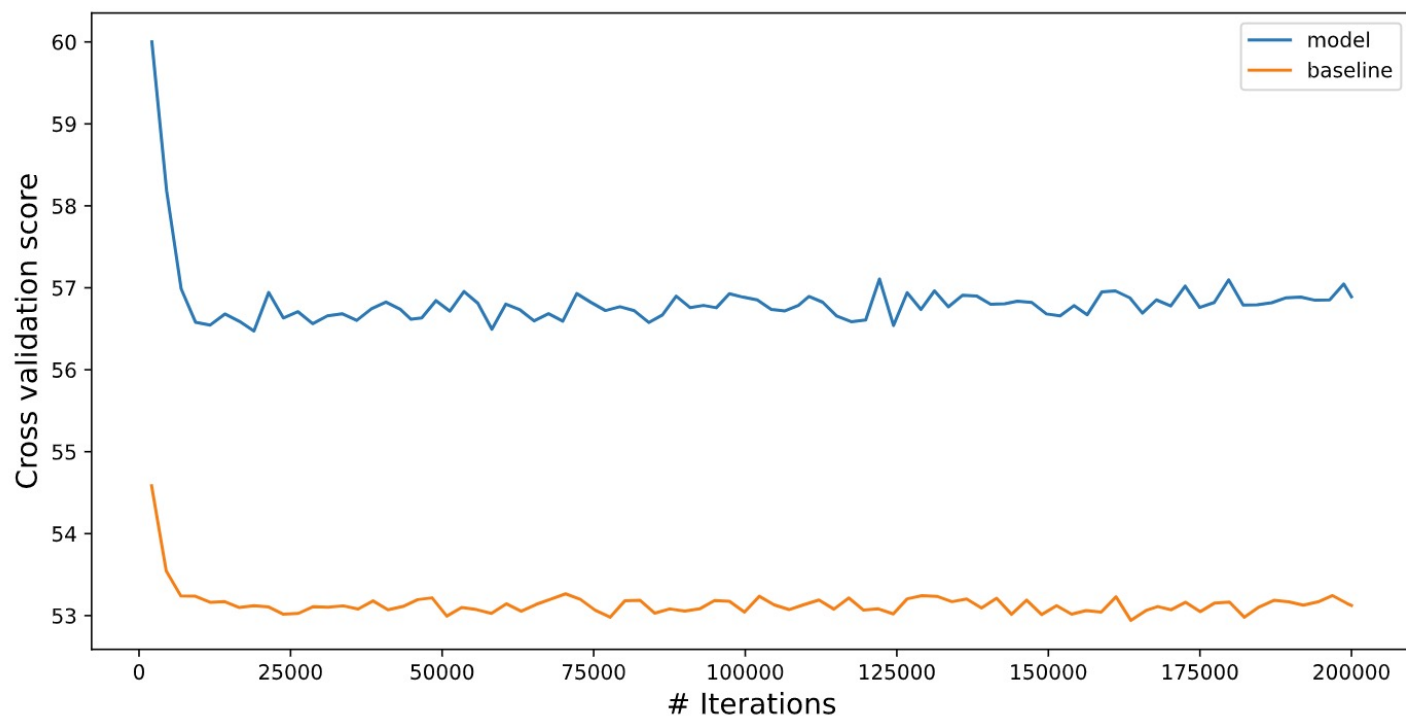

B

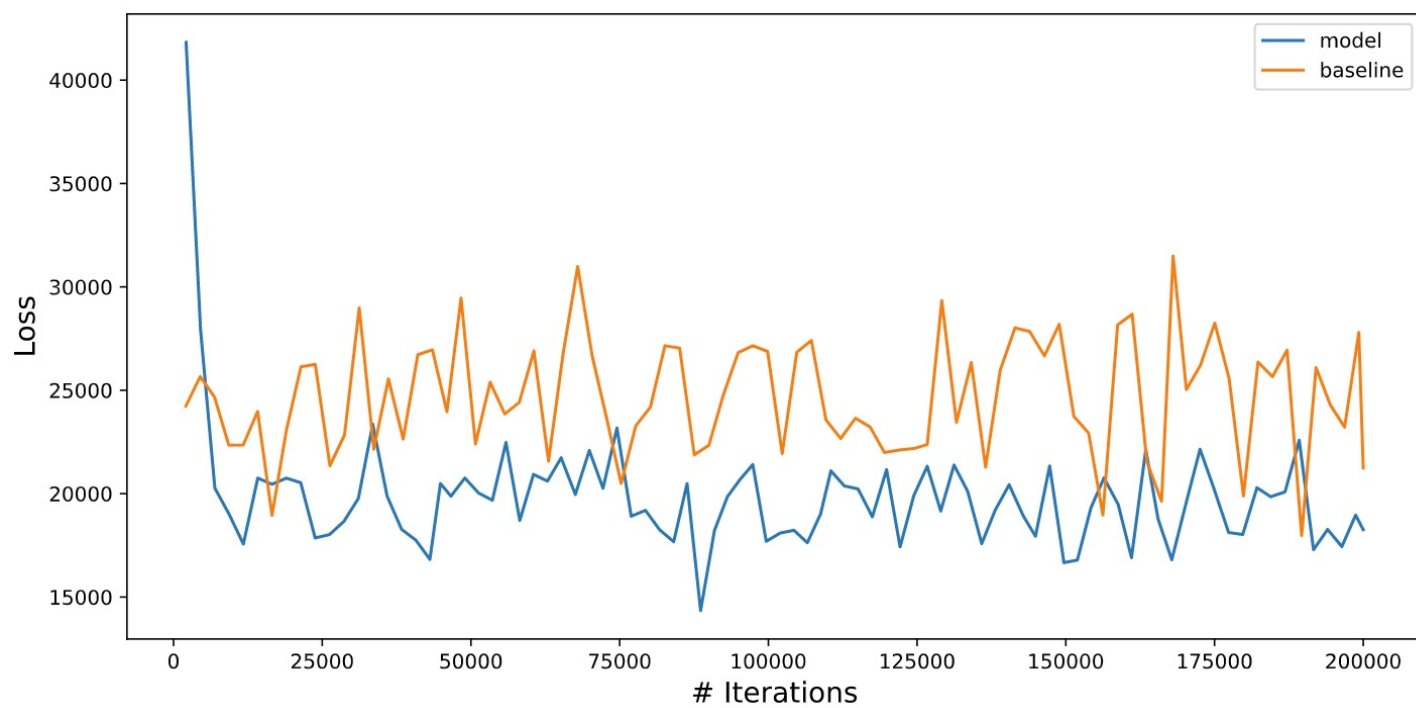

Supplement: Supplementary file 3 — Additional file 3: Supplementary Fig. 3. Rumen microbiome convergence summaries. The A) cross-validation score and B) loss plots are shown for the Songbird multinomial regression model used to compute log-fold changes in rumen microbial abundance relative to health status. The blue line represents our model computing log-fold changes with respect to health status; the orange line represents the null or baseline model demonstrating log-fold changes due to random chance. [file 12917_2022_3500_MOESM3_ESM.pdf]
